# COVID-19, HIV, and Cryptococcal Meningitis Coinfections with Abnormal Laboratory Findings

**DOI:** 10.1155/2023/2868290

**Published:** 2023-11-21

**Authors:** Mina Aghamali, Abdolhassan Kazemi, Mohammad Asgharzadeh, Hossein Samadi Kafil

**Affiliations:** ^1^Student Research Committee, Tabriz University of Medical Sciences, Tabriz, Iran; ^2^Research Center for Pharmaceutical Nanotechnology, Tabriz University of Medical Sciences, Tabriz, Iran; ^3^Department of Parasitology, Faculty of Medicine, Medical Philosophy and History Research Center, Tabriz University of Medical Sciences, Tabriz, Iran; ^4^Biotechnology Research Center, Tabriz University of Medical Sciences, Tabriz, Iran; ^5^Drug Applied Research Center, Faculty of Medicine, Tabriz University of Medical Sciences, Tabriz, Iran

## Abstract

**Background:**

Severe acute respiratory syndrome coronavirus 2 (SARS-CoV-2) was first introduced in China in 2019, and it has rapidly spread all around the world. *Cryptococcus neoformans* is the leading cause of fungal meningitis in human immunodeficiency virus- (HIV-) infected patients. A variety of laboratory tests have been introduced for rapid diagnosis of meningitis.

**Methods:**

Here, we report a case of coinfection with COVID-19 and cryptococcal meningitis in a HIV-positive patient with abnormal laboratory findings. In this case, COVID-19 was positive by polymerase chain reaction (PCR) and computerized tomography (CT) scan diagnosis. Cryptococcal antigen testing of CSF was negative, whereas India ink staining and cerebrospinal fluid (CSF) culture confirmed the presence of *C. neoformans*.

**Results:**

Although the patient was in a critical stage of illness, serum and CSF levels of procalcitonin were abnormally low, within normal limits. On the other hand, although initial lumbar puncture had showed elevated protein level, the repeat CSFs presented remarkably reduced protein levels. Our findings indicate that despite COVID-19 infection, procalcitonin level may remain normal in HIV-associated cryptococcal meningitis, and findings of an apparently normal procalcitonin level should not exclude the possibility of infection. Also, antigen testing may present false-negative result, and it should not be the sole laboratory method for diagnosis of infectious meningitis. Consequently, CSF culture and staining is recommended, even when antigen testing of organism is negative and CSF profile is unremarkable.

**Conclusion:**

Laboratory information should be combined with a good understanding of clinical manifestations of patient to determine if meningitis is present and confirmed COVID-19 should not ignore possibility of other infections for consideration.

## 1. Introduction

SARS-CoV-2 (COVID-19) is an RNA virus from family *Coronaviridae* and genus *Betacoronavirus* that emerged in Wuhan, China, during the year 2019, and infection developed by this virus is called COVID-19 (coronavirus disease 2019) [1, 2]. This infection caused wide range of disorders and widely studied during the past year [3, 4]. It has different clinical manifestations from lung, blood clotting, and liver to kidney disorders [5]. *Cryptococcus neoformans* stands out as a leading cause of fungal meningitis in immunocompromised patients, particularly HIV-infected individuals [6]. The life-threatening consequences of delayed diagnosis necessitate rapid laboratory tests for prompt initiation of appropriate therapy. In patients suspected to infectious meningitis, lumbar puncture will help in arriving at the correct diagnosis [7]. Also, analysis of cerebrospinal fluid (CSF) in terms of glucose and protein levels, white blood cell (WBC) count, CSF culture, and staining is one of the most important laboratory tests for identifying or ruling out the causative organism [7]. In children and adults, WBC count of normal CSF is 0-5 cells\mm^3^, and values more than this norm were considered as an abnormality [7]. Also, under normal circumstances, glucose and protein concentrations of CSF include 45-80 mg/dL and <45 mg/dL, respectively [7]. Any change in these ranges is a reflection of disease and helps for rapid diagnosis. The other alternative is serum/CSF cryptococcal antigen test. Normal CSF is clean and devoid of any organism [7]; therefore, pathogen antigen detection provides excellent and rapid results for diagnostic laboratories [8]. Along with the above-mentioned tests, blood studies including complete blood count and blood culture and evaluation of biomarkers such as C-reactive protein (CRP) and procalcitonin (PCT) have shown great impact on early detection of meningitis. Here, we present a case of COVID-19 and cryptococcal meningitis in a HIV-positive patient with abnormal laboratory findings.

## 2. Case Report

The study was approved by the Institutional Review Board of Tabriz University of Medical Sciences (IR.TBZMED.REC.1398.1277) and performed in accordance with the principles of the Declaration of Helsinki. Written informed consents were obtained.

**Figure 1:**
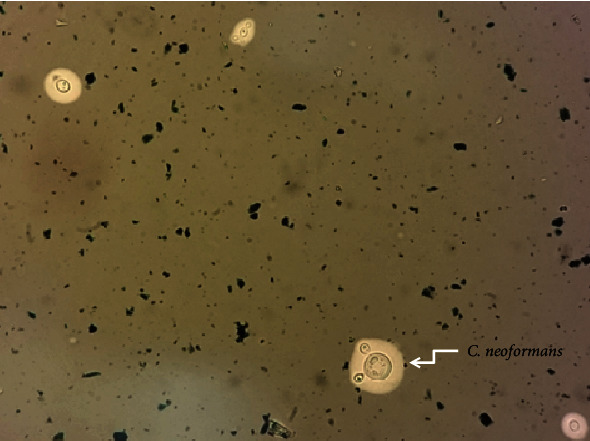
India ink preparation showing capsules of *Cryptococcus neoformans.*

A 28-year-old man admitted for malaise, fever, and headache which were present for the previous seven months. Recently, increased cough and chest pain were added. Past medical history of the patient was notable for hepatosplenomegaly, generalized lymphadenopathy, pleural effusion, and hemoptysis. Also, pancytopenia, diffuse large B-cell lymphoma (DLBL), and plasma cell dyscrasia had been documented over three hospitalizations, in which patient had discontinued treatment. The patient was hospitalized in infectious disease ward. Mental status was normal, and skin and ocular involvement were not found at presentation. A full blood count showed low levels of WBC (1.9 × 10^3^ cells/mm^3^), RBC (3.11 × 10^3^ cells/mm^3^), platelet (86 × 10^3^ cells/mm^3^), and hemoglobin (6.2 g/dL). Serum procalcitonin was 0.068 ng/mL, but CRP was elevated to 67.8 mg/L. RT-PCR (real-time polymerase chain reaction) for COVID-19 (Pishtaz Co. kit, Iran) was positive and CT scan showed lung involvements. Magnetic resonance imaging (MRI) of the patient demonstrated multiple hypersignals, and metabolic diseases such as Wilson, poisoning with carbon monoxide, and drug reaction were in differential diagnostic list. Lobar pneumonia at the right lung was detected by chest X-ray. During admission, dizziness, vomiting, sweating, and hyponatremia were added to clinical spectrum. Initial lumbar puncture showed elevated opening pressure, WBC count 50 cells/mm^3^ (70% segmented and 30% lymphocytes), glucose level 35 mg/dL, protein level 71 mg/dL, lactate dehydrogenase (LDH) level 64 mg/dL, and adenosine deaminase (ADA) level 10.9 U/L. Procalcitonin level of CSF was 0.084 ng/mL. Although cryptococcal rapid antigen testing (Coris BioConcept, Belgium) of CSF was negative, India ink staining of CSF sediment revealed *C. neoformans* (Figure 1). The diagnosis of cryptococcal meningitis was confirmed by isolation of *C. neoformans* from CSF culture (400 cells/mm^3^). Niger seed agar medium (Merck, Germany) was used for culture and confirmation. Brown color colonies were considered as *C. neoformans.* The CSF Wright test or Coombs wright test was negative. Blood and urine cultures remained negative during admission. The patient was initiated on intravenous remdesivir and amphotericin B. The result of an HIV antibody testing was positive (291.03 IU/L). The CD_4_ count was performed which turned out to be 11 cells/mm^3^. Repeat lumbar punctures were performed, presenting remarkably reduced protein level of CSF to 3 and 2 mg/dL. Fluconazole and tazocin were added to treatment options. Unfortunately, the patient had extensive cryptococcal involvement of meninges and expired within 5 days after diagnosis.

## 3. Discussion

Cryptococcal meningitis is primarily an AIDS-defining disease, although cases of infection occur in HIV-negative individuals. Coinfection with COVID-19 can provide necessary context to develop such infections. A delayed diagnosis may lead to severe long-term consequences or even death, while early detection of causative organism and appropriate therapy can be lifesaving. Therefore, a prognostic biomarker is necessary to reduce delayed results and mortality rate [9]. Among biomarkers, PCT appears to be one of the most sensitive and specific predictors of infections. It is usually secreted in trace amounts by thyroid gland of healthy individuals (<0.1 ng/mL) and increases sharply in various infectious disease which may reach to over 100 ng/mL [10–12]. Also, serum PCT level is associated with severity of disease, providing opportunity for monitoring of treatment [13]. In the current case, although patient was in critical stage of illness, both serum and CSF levels of PCT were abnormally low, within normal limits (0.068 and 0.084 ng/mL, respectively). The current finding is not consistent with previous reports. Several studies in literature have reported elevated PCT level in infectious diseases. Although rate of elevation in bacterial infections is more than viral and fungal disease, at least slight increase must be seen in critical fungal disease. The reason why the level of PCT was low remained unclear. One possible explanation is the compromised immune system of the patient. As patients with AIDS are unable to mount an adequate inflammatory response, PCT level may not be a reliable marker for diagnosis of meningitis. On the other hand, it may be attributable to medical history of our patient. Result of cryptococcal antigen testing is the other issue to be considered in this case. In our patient, cryptococcal antigen testing of CSF was negative, whereas India ink staining and CSF culture confirmed the presence of yeast cells. There is consensus that culture for growth of etiologies often takes a few days to obtain a final result and a rapid test such as antigen detection is more preferable. Also, predictive value of antigen testing has been confirmed by studies, and CDC (Centers for Disease Control and Prevention) has found it more advantageous over traditional methods [14]. However, our finding indicates that the antigen testing of etiologies, although sensitive and specific alternative, should not be the sole laboratory method for diagnosis of infectious meningitis. In people with cryptococcal meningitis, the CSF often shows an increased WBC count and protein level along with reduced glucose level [7], while in COVID-19 and HIV-infected patients, results may differ. In this case, initial lumbar puncture showed elevated protein level, and the repeat CSFs presented remarkably reduced protein levels. Regarding to our case, routine CSF analysis is not reliable predictor in HIV-positive patients, particularly in individuals with underlying hematological disorders such as pancytopenia.

## 4. Conclusion

COVID-19 infection can provide an opportune for other infection in immune-deficient patients. Our findings provide evidence that PCT level may remain normal in COVID-19 and HIV-associated cryptococcal meningitis. PCT, as useful marker for monitoring of infection, should complement other laboratory parameters rather than replacing, and findings of an apparently normal PCT level should not exclude the possibility of infection. Since the pathogen antigen testing present false-negative result, it should not be the sole laboratory method for diagnosis of infectious meningitis. Consequently, CSF culture and staining is recommended, even when antigen testing of organism is negative and CSF profile is unremarkable. Protein and glucose levels of CSF do not always match with diagnostic criteria; therefore, diagnosis based on only one of these tests may result in delayed or wrong measurements, needless to say that laboratory information should be combined with a good understanding of clinical manifestations of patient to determine if meningitis is present. COVID-19 can change total face of infections and biomarker levels in infected patients which may confuse us in the future.

## Data Availability

All data and analysis results related to this study are available by sending a request to the corresponding author.
